# U.S. Children and Youth’s Physical Activities Inside and Outside of School PE: 1985 vs. 2012

**DOI:** 10.3390/ijerph18020398

**Published:** 2021-01-06

**Authors:** Xiaochen Zhao, Weimo Zhu, Zezhao Chen, Sicong Ren, Xiong Qin

**Affiliations:** 1 Department of Sports Science and Physical Education, Tsinghua University, Beijing 100084, China; zxc17@mails.tsinghua.edu.cn; 2 Department of Kinesiology and Community Health, University of Illinois, Urbana, IL 61801, USA; weimozhu@illinois.edu (W.Z.); zchen106@illinois.edu (Z.C.); sicongr2@illinois.edu (S.R.)

**Keywords:** physical education, activity ranking, youth sport, national studies

## Abstract

The purpose of this study was, by taking advantage of the rich data from two U.S. national fitness surveys, to examine the physical activity (PA) students engaged in, both inside and outside school physical education (PE), determine if there are differences by grade, sex, and weight status, and if there was a change between 1985 and 2012. The data from the 1985 National Children and Youth Fitness Study (NCYFS) and the 2012 NHANES National Youth Fitness Survey (NNYFS) were matched, merged (*N* = 6178, 3107 boys and 3071 girls), and analyzed. It was found that basketball remained the most popular PA inside school PE across both surveys. Swimming was the most popular PA outside of school PE in 1985, but was replaced by running in 2012. Although PA taught and promoted inside of school PE and that such PA practiced outside were moderately correlated across the surveys, some disconnections were noticed. The impact of grade, sex, and weight status on PA preference and participation was also confirmed. What is needed is to the design and integration of more lifelong and individual PAs in future school PE curricula and school and community children and youth sport and PA programs.

## 1. Introduction

Obesity has become a significant worldwide threat to public health [1,2,3]. In the U.S., for example, obesity in children and youth has become an increasingly severe issue, and physical activity (PA) shows a downward trend during adolescence [4]. Recent estimates indicated that obesity prevalence was 13.9% among 2- to 5-year-olds, 18.4% among 6- to 11-year-olds, and 20.6% among 12- to 19-year-olds. Childhood obesity is also more common among certain subpopulations, e.g., African-American and Hispanic children and youth [5]. For that reason, many experts and researchers have been seeking ways to address childhood obesity, including the introduction of national PA guidelines and fitness assessment, educational programs, and determining the time spent and intensity of PA among children and youth, while examining the main correlates affecting obesity from various aspects of society [1,2,3,6,7,8,9,10]. The 2nd set of PA Guidelines issued by the United States Department of Health and Human Services in 2018 recommended 60 min or more per day of PA for school-aged children and adolescents [11]. PA can not only help improve children’s physical fitness and health [12], but also help improve their mental health. It worth noting that some studies have confirmed school’s PE requirement seems to improve children’s PA [13], but obese children still performed fewer PA at school than other children [14]. Besides, adding one PE lesson to school daily will effectively reduce sedentary behavior in children [15]. The repertoire of choices, the more likely students will remain committed sports participants when moving from youth to adulthood [16]. Perhaps the most important thing is to create more PA participation opportunities for children and youth so that they can learn and enjoy them [17]. 

Since children and youth spend a large proportion of their time in school, school physical education (PE) has a momentous effect on them to promote lifelong health concepts and to cultivate good exercise habits [16,18]. One question naturally raised is what kind (e.g., sport or leisure activities) and how many different (e.g., a variety of activities or only a few of them) PA should be taught in school? A student is typically exposed to 12 different activities in PE class per year, and girls are slightly more diverse than boys [19]. In particular, persistent participation in PA in school will increase the probability of a higher level of PA in later life [6,20], and the single strongest predictor of people’s later-life PA was whether they played a varsity sport in high school [21]. Furthermore, the school environment and PE are important ways to encourage their health-related behaviors and to engage in daily PA as they age. 

Nevertheless, as of 2000, only 49% of schools offered intramural sports [22]. In order to improve this situation, the Committee on Prevention of Obesity in Children and Youth recommended that intramural sports be more widely introduced within schools, to meet the needs of students who lack skills or sufficient confidence to participate in sports. The committee also suggested that such sports projects should become a vital component of both in and out of school [23]. Encouraging systematic or frequent PA at a young age, whether through school sports or club opportunities, might be the best investment in long-term PA [21]. People’s lifelong PA behaviors are inseparable from the sports they participated in during their youth. For long-term diversified PA, appealing to more students to participate in school or out-of-school PA is especially important. At present, there are several studies indicating that sport participation that involves family and peers is a positive factor that may help youth to develop regular PA habits through socializing [20,24,25,26], and proved from various aspects that sports companions are an important factor affecting youth participation in sports activities, especially after-PE activities that may directly affect the formation of lifelong sports habits [25]. 

Meanwhile, there is still much room to improve in terms of what to teach and how to teach PA for children and youth, considering that childhood and adult PA were weakly correlated (*r* = −0.08–0.14) and total weekly PA in childhood did not at all predict adults’ PA participation well [27]. Furthermore, in addition, in public schools, there has been a steady decline in school PA, but an increase in childhood obesity since the 1970s [28,29,30]. What is the role of PA taught in school and practiced outside school in the current childhood obesity epidemic? Is there any difference between boys and girls, normal weight and overweight/obese children in terms of the type of PA engaged in outside of school? Answering these questions should shed some light on how much PA should be taught in school and help improve children’s PA participation outside of school. 

Fortunately, two U.S. children and youth fitness studies, one in 1985 known as the National Children and Youth Fitness Study (NCYFS), and another one in 2012 known as NHANES National Youth Fitness Survey (NNYFS), in which children and youth’s PA taught or organized in school and participated outside school were also investigated. By taking advantage of the rich data from these two U.S. national fitness surveys, the purpose of this study was to examine the PA students engaged in, both inside and outside school PE, determine if there are differences by grade, sex, and weight status, and if there was a change between 1985 and 2012.

## 2. Materials and Methods 

### 2.1. Data and Participants

The 1985 NCYFS was the first study of the fitness of U.S. youth based on a nationally representative sample. The study included 140 schools in 19 states, 10,275 students in grades 5–12 (5140 boys and 5135 girls), and among those, 8800 (4539 boys and 4261 girls) completed both fitness testing and a PA survey. For the PA survey, students were first asked to complete some personal information, including age, sex, grade, and the frequency of participation in PE courses and changing clothes/washing time during the past year. Then, they were given a list of 86 PAs and asked to check which, if any, of them if they participated in their PE classes during the past year. Finally, they were given the same PA list again and asked to check which ones, if any, they participated in outside PE, including any after-school (in community organizations, at home, and in the neighborhood) during the past year.

Selected from an independent sample of dwelling units within the U.S. 2012 National Health and Nutrition Examination Survey (NHANES), NNYFS was conducted in 2012 to collect data from a total of 2065 children and youth ages 3–15. Of those, 1640 (79.4%) were interviewed, and 1576 (76.3%) were physically examined. The survey included an extensive array of daily activities, leisure-time activities, and sedentary activities, including weekly frequency, duration, and intensity. The questions were asked in the home by trained interviewers using the Computer-Assisted Personal Interviewing system (CAPI). For school-related PA, the students were asked if they participated in any of the 19 specific sport or PA clubs, plus a choice of “Other (Specify)”; for no school PA, they were asked if they participated in any of the 31 PA, plus a choice of “Other (specify)”. 

The age groups of the participants between two surveys were matched. In order to facilitate comparison and the data analysis across years, only children and youth aged 10–15-year-old and grades 5–10 were included, which ultimately led to a sample size of 6178 (3107 male and 3071 female) from NCYFS and 759 (379 male and 380 female) from NNYFS. 

### 2.2. Measurements

PA questionnaires. There were some differences in PA questionnaires between 1985 NCYFS and 2012 NNYFS. The NCYFS questionnaire used the same 86 specific PAs in both inside and outside the PE survey and asked the students if they participated in them in the past year. For the school PE, NNYFS asked only if the students participated in any 19 sports or PA clubs, plus one “Other (Specify)” without a specific timeframe, and for the outside PE, NNYFS used only 31 specific PA, plus “Other (Specify)” and asked the students if they participated in them during the past 7 days.

To make the comparison, some adjustments were made in 1985 NCYFS PA: (a) “Dance” included ballet, jazz, modern dance, ballroom(cotillion) dance, disco/popular music, folk/square dance, and other vigorous dances; (b) “Gymnastics” included apparatus (with equipment), free exercise, rhythmic, tumbling; (c) “Football” included football (tackle) and football (touch or flag); (d) “Play games” included hopscotch, king of the hill/capture the flag, red rover, spud, tag, tug-of-war; and (e) “Backyard games” included climbing ropes/monkey bars, croquet/miniature golf, Marco polo/underwater games, paddleball, punch ball, tetherball. After the adjustment, 1985 NCYFS’ PA list was reduced to 61 for both inside and outside school PE, and the PA number of 2012 NNYFS remained the same.

Body mass index (BMI). The weight status of the student was determined using the CDC’s BMI standard [31]. The 2012 NNYFS data already included each student’s BMI percentiles computed based on the data from the U.S. national surveys from 1963–1965 to 1988–1994: Underweight (BMI <5th percentile), Normal weight (BMI 5th to <85th percentile), Overweight (BMI 85th to <95th percentile), and Obese (BMI ≥95th percentile) [32]. For 1985 NCYFS data, we calculated the BMI for each student using the same standard.

### 2.3. Statistical Analysis 

The data from two surveys were merged first and, to maintain their national population representations, this study used the weights recommended by the analysis process of the surveys. The descriptive statistics for all key variables were analyzed by survey first. The preferred PAs were ranked based on the number of the student participation by survey, sex, and obesity status. By computing the Spearman rank correlation coefficients, the relationship between PA participation rank in and outside of school were then examined by the survey and across the surveys, respectively. The degree of the correlation was evaluated using the following absolute criteria [33]: 0.00–0.19 = “No correlation”, 0.20–0.39 = “Low correlation”, 0.40–0.59 = “Moderate correlation”, 0.60–0.79 = “Moderately high correlation”, ≥0.80 = “High correlation”.

## 3. Results

Table 1 summarizes the demographics of participants in the 1985 and 2012 surveys. One of the sad, yet expected, findings was that, compared with their counterparts in 1985, the students in 2012 were about 10% heavier. As a result, their BMIs also increased significantly, especially for boys in Grade 8. 

Table 2 summarizes PA participation in school in the 1985 survey by total, sex and BMI. Overall, the most popular PA activity in school was basketball among U.S. students (83.6%) in 1985, followed by jogging (distance running) (73.4%), baseball/softball (71.5%), calisthenics/exercises (71.4%), volleyball (71.3%), play games (66.9%), soccer (63.8%), track relays (59.2%), kickball (58.6%), football (56.4%), backyard games (56.2%), running sprints (54.9%), gymnastics (54.7%), dance (44.6%), jumping rope (43.8%), track and field (not running) (29.6%), field hockey/street hockey (27.3%), stickball (21.5%), badminton (20.2%), and weightlifting or training (19.4%), respectively. 

There was little difference between boys and girls in terms of PA participation in school PE. Of the top 20 most frequently PA by boys and girls, there were only two significantly different ones; boys reported more wrestling (ranked 20th), whereas girls reported more fast walking (20th). There seems also limited differences among BMI subgroups in terms of the PA they participated in, e.g., basketball remained the highest-ranked sport among all groups. 

Table 3 summarizes PA participation outside of PE in the 1985 survey by total, sex and BMI. Swimming/diving was the most frequently reported PA outside of PE by the students (85.7%), followed by bicycling (82.8%), basketball (76.9%), play games (75.2%), baseball/softball (74.4%), football (70.7%), roller skating (69.2%), backyard games (68.5%), jogging (distance running) (61.5%), Frisbee (57.7%), fishing (56.5%), dance (55.9%), kickball (55.4%), volleyball (54.7%), soccer (53.00%), ping-pong (table tennis) (46.9%), gymnastics (45.0%), jumping rope (44.8%), track relays (43.3%), and calisthenics/exercises (42.6%), respectively. 

Again, there was little difference between boys and girls in terms of their favored PA outside of PE. Of the top 20 popular PAs ranked by students, 16 of them were participated in by both boys and girls. Weightlifting or weight training (ranked as 16th), running sprints (18th), wrestling (20th) were among the top 20 reported for boys, while fast walking ranked (18th) by girls. Weight status of the students seemed to have an impact on the preference of PA participation. For example, the under- and normal-weight groups ranked bicycling first, while overweight and obese groups preferred swimming/diving. Among the top 10 PA by sex subgroups, boys and girls in the normal-weight group preferred jogging, while among those in the obese group, more boys chose Frisbee, while more girls selected volleyball.

The top part of Table 4 summarizes the correlations between the 1985 rankings of PA in and outside PE by total, grade, sex, and weight status. Overall, there was a moderately high correlation (ρ = 0.702) between PA ranks participated inside and outside of school PE. A grade difference was noticed, however; 6th-grade normal-weight girls had the highest overall correlation (ρ = 0.785), and 9th-grade underweight girls had the lowest correlation (ρ = 0.305). There seemed a trend; as the grade level increased, the degree of relevance of activities inside and outside PE decreased. Compared with the lower-grade students, students in higher grades chose more sports-related PA.

Table 5 summarizes sports or PA club’s participation in school in the 2012 survey by total, sex, and BMI. Again, basketball (18.0%) was still the most popular club participation, followed by soccer (12.0%), baseball (10.1%), football (9.3%), track and field (7.0%), volleyball (5.6%), running (4.2%), dance (3.2%), cheerleading (2.9%), swimming/diving (1.8%), tennis (1.7%), golf (1.6%), gymnastics (1.6%), wrestling (1.6%), lacrosse (1.4%), field hockey (1.2%), trampoline (0.6%), martial arts (0.4%), Frisbee (0.3%), fast walking (0.2%), respectively. Some sex difference was noticed, e.g., no boys chose cheerleading or martial arts, while no girls joined lacrosse, Frisbee, wrestling, or bocce ball. For the underweight group, dance had the largest number of participants, and the other three weight groups still chose basketball as their most popular choice. Compared with the normal-weight group, the top ten PA for the overweight and obese groups included tennis, wrestling, and swimming/diving, respectively. In addition, obese boys preferred to join field hockey instead of wrestling, and surprisingly, football was ranked a higher PA by the obese girl group.

Table 6 summarizes PA participation outside of PE in the 2012 survey by total, sex and BMI. Running (26.9%) became the most popular PA, followed by basketball (26.4%), fast walking (22.6%), bicycling (21.6%), football (12.6%), soccer (12.5%), backyard games (12.5%), baseball (11.4%), dance (10.6%), play games (9.5%), swimming/diving (8.3%), aerobics (7.2%), volleyball (4.4%), skateboarding (4.3%), jumping rope (4.2%), trampoline (4.2%), tennis (3.5%), scooter riding (3.3%), hiking (3.2%), track and field (3.0%), respectively.

For the top 20 PA participation by sex, wrestling and Frisbee were more popular among boys, and gymnastics and cheerleading among girls. There seemed an “interaction” between sex and weight status in terms of choosing PA. For example, among obese boys, the top five PA participation were running, basketball, bicycling, football, and walking, respectively, while the top five PA for obese girls were walking, bicycling, dance, basketball, and running, respectively (see also Table 6). 

The bottom part of Table 4 summarizes the correlation between the 2012 rankings of school sport/PA clubs and PA outside PE participation by total, grade, sex, and weight status. As expected, the correlation (ρ = 0.556) was lower than in the 1985 survey since different survey questions were asked in 2012. It was noticed that the correlations between boys in all grades were much higher than the correlations among girls. Moreover, the overweight group had the lowest correlations among both boys and girls.

Finally, Table 7 summarizes the correlations of PA participation between 1985 and 2012. The overall rank correlation between the 1985 PA participation in school PE and 2012 sport/PA clubs participation in school was 0.726, and between 1985 and 2012, PA outside PE was 0.680, i.e., both had a moderately high correlation. By examining the correlation by grade, sex, and weight status, however, some large variations were noticed. For example, for Grade 5, obese girls, the correlation between 1985 and 2012 in school PA participation was only −0.200, and for Grade 9, obese boys, the correlation between 1985 and 2012 PA participation outside PE was 0.631.

## 4. Discussion

The primary purpose of this study was, by taking advantage of the rich data from two U.S. national PA surveys, to examine what PA were students engaged in, both in and outside of schools, to learn if there is a difference by grade, sex, and weight status, and what changes existed between 1985 and 2012. This is also the first study focusing on children and adolescents’ PA patterns and tendencies both inside and outside school across years. Inside of school, either in PE or through sport or PA clubs, basketball remained the most popular PA both in 1985 and 2012, and swimming/diving had the most participation outside of PE in 1985, but it was replaced by running in 2012. The overall correlations between in-school PA and the PA outside school, PE was higher in 1985 than in 2012, but the difference could have been caused by two different questionnaires that were used in the 2012 survey. Compared with overweight and obese groups, the selection of PA in normal-weight groups was more similar between 1985 and 2012.

There were some major changes between 1985 and 2012 in terms of PA participation inside and outside of school. Although basketball, running, baseball, volleyball, soccer, football, gymnastics, dance, track and field, and hockey were still in the top 20 inside school PA in 2012, some new PAs jumped to the top of the ranking list compared with 1985, including cheerleading, swimming/diving, tennis, and wrestling. Overall, 40% of PAs changed in the top 10 PAs and 50% changed in the top 20 PAs between the two surveys. In 2012, again because the focus was on the sports and PA clubs organized by the schools, many team sports jumped to the top-ranked PA, such as football and soccer. Clearly, the more competitive sports were still the center of school PE and athletic programs in U.S. [19]. 

For the PA outside school PE, 8 PAs were added to the top 20 rankings in 2012, including fast walking, aerobics, skateboarding, trampoline, tennis, scooter riding, hiking, and track and field. Especially noteworthy was that new PAs such as trampoline and bocce ball were included in the 2012 questionnaire, but 42 PAs were excluded, such as fencing, yoga, and squash. Besides, some PAs ranking left behind by the rest of the others both in 1985 and 2012, which may be affected by seasonal factors and regional differences, making the development of these projects not optimistic. The enthusiasm of students to participate could also be affected (e.g., ice skating and ice hockey).

Some changes in the “interaction” between sex and PA preference were also noticed. In 1985, the distribution of sex ratios in various PAs participation was more balanced, and there were only limited differences in terms of the boy-girl percentages in most PA participations. In 2012, however, there seemed to be more sex differences in terms of preferences for a specific PA participation. For example, about 70–90% of boys chose baseball, rugby, golf, and others, while nearly 70% of girls chose volleyball, dancing, cheerleading, and gymnastics. The sex and PA interaction, i.e., boys were more inclined to choose team sports (e.g., football, basketball, and baseball) while girls preferred to participate in lifelong, individual PAs (e.g., swimming, walking, and dance), which has also been reported in other studies [26,34,35,36]. Therefore, school PE curricula and programs should take the sex difference into consideration when choosing PAs and design and promote more “sex friendly” PAs in both school and community settings [37].

Lifelong, individual PAs becoming more popular was another noticeable finding when comparing the 1985 and 2012 surveys. For example, among the top 20 extracurricular activities reported by the students in 2012, participation in team sports was far lower than the lifelong, individual PAs. As a result, there seems to be a disconnection between sports/PA clubs promoted inside of school and PAs preferred by the students outside of school. The future school PE curriculum should recognize the lifelong PA trend and teach/promote more lifelong, individual PAs, such as walking, running, yoga, etc. 

This is the first study to examine the preference of PA among different groups (e.g., by age and sex), but especially examined the insight of PA choices by children and youth with different BMI. The impact of one’s weight status on PA preference and selection was another interesting finding of this study. Among the top 20 PAs inside of school PE in 1985, for example, compared to the normal-weight students, the obese students preferred “weightlifting or training” more than “fast walking,” and furthermore, they ranked jogging higher than their normal-weight counterparts. However, jogging was ranked higher (9th) by the normal-weight students outside of school PE than the obese students (14th). Some differences were noticed in the 2012 survey, in which normal-weight students participated in more running clubs, while obese students were more willing to participate in walking. In addition, in the top-20 ranked PAs, the normal-weight students preferred to participate in track and field, tennis, and scooter riding, while wrestling, gymnastics, and Frisbee were more popular among obese students. It was noticed that, due to the interest in weight management, more obese students participated in aerobics for their PA outside of school PE. Considering that childhood obesity is a national and international health problem, school PE curricula, programs, and specific PAs taught should be targeted to obese students for their lifelong effort in weight management. If the school curriculum can determine what kind of PA the students in different groups prefer, they could change the curriculum more specifically. Besides, autonomy is one of the crucial triggers for children to participate in PE [38].

There were several sports-related changes made between the 1985 and 2012 surveys. As a good aerobic exercise, running has always been favored as a school PA, and its ranking has always been relatively high [39]. That was also true in the 1985 PA inside of school PE, but not PA outside of PE. In 2012, however, running became the most popular PA outside of school PE, but its inside school ranking dropped to seventh. For inside school sport or PA clubs, students preferred more team sports, such as basketball, soccer, baseball, football, etc. That is understandable since team sports are more social and fun for the students, while running is a much easier to do PA individually in both the community and home settings. To some extent, the individual PAs have a tendency to develop into a lifelong PA habit more than team sports. The promotion of lifelong and individual PAs within the school, as well as popular team sports, should be areas to explore. 

One of the observed interesting changes between the 1985 and 2012 surveys was the ranking of swimming. In 1985, it was ranked only 26th in school PE, but jumped to 10th in preferred school sport club in 2012. That change may be due to more schools in the U.S. that now have swimming pools and, as a result, are able to provide swimming lessons and activities inside of schools. Some studies demonstrated that secondary-school students with a larger number of outdoor facilities available had almost three times higher odds to participate in daily PA during recess compared with students with fewer facilities [40,41].

While the findings of this study provided, for the first time, PA participation inside and outside of school PE between 1985 and 2012, a few limitations should be acknowledged. First, different survey questionnaires were used in 1985 and 2012. As a result, some information generated may be limited and may not be representative, e.g., PA inside and outside of school PE may now mean different things. Second, when matching PAs across years, some useful information may have been omitted (e.g., play games and backyard games were all grouped into “Play games”), and some items may be affected by seasonal factors, which, in return, may affect the ranking of a specific PA. Third, despite the fact that both surveys employed nationally representative samples, the studies were conducted several years ago and may not be able to completely represent current levels of PA participation in the U.S. today. Finally, PA information in both surveys was self-reported and may not reflect the true PA participation of the students due to the nature and limitation of their recall. 

## 5. Conclusions

In conclusion, using the rich information from two U.S. national fitness surveys, children and youth’s PA participation inside and outside of school were examined. While some PAs, like basketball, maintained the same popularity in both surveys, some changes were observed in PA participation both inside and outside of school PE. This is also the first study to examine the correlation between PA taught and promoted inside of school PE and those participated outside of school PE, and some disconnections were noticed although, overall, a moderate correlation was found between them. The impact of age, sex, and weight status on the selection and participation in PA was also confirmed. Compared with the normal-weight groups, obese children and youth had different preferences to PAs (e.g., wrestling and Frisbee), and boys preferred more to choose team sports, while girls inclined to participate in individual PAs. There also seemed a trend that, as the grade level increased, the degree of relevance of activities inside and outside PE decreased. What is needed is the design and integration of more lifelong and individual PAs in future school PE curricula and sport and PA programs.

## Figures and Tables

**Table 1:** Demographics of the students in 1985 and 2012 surveys (mean ± SD).

Grade	Variable	1985			2012		
		Total	Boys	Girls	Total	Boys	Girls
5	*N*	1,499,781	712,022	787,759	4,357,681	2,186,984	2,170,697
	Height (cm)	143.79 ± 8.19	143.36 ± 8.2	144.18 ± 8.16	143.45 ± 7.48	143.29 ± 7.54	143.63 ± 7.42
	Weight (kg)	37.61 ± 9.57	37.43 ± 10.2	37.79 ± 8.96	42.19 ± 13.68	43.59 ± 15.43	40.78 ± 11.36
	BMI	18.1 ± 3.45	18.15 ± 3.6	18.06 ± 3.3	20.17 ± 5.08	20.83 ± 5.72	19.51 ± 4.24
6	*N*	3,179,254	1,630,639	1,548,616	3,787,613	1,685,869	2,101,744
	Height (cm)	148.4 ± 7.49	147.42 ± 7.32	149.44 ± 7.52	151.04 ± 7.48	150.97 ± 8.08	151.08 ± 6.95
	Weight (kg)	42.1 ± 9.48	40.96 ± 8.74	43.29 ± 10.07	47.41 ± 13.8	48.39 ± 15.06	46.56 ± 12.67
	BMI	18.97 ± 3.25	18.72 ± 2.99	19.24 ± 3.48	20.51 ± 4.75	20.90 ± 5.05	20.19 ± 4.46
7	*N*	3,347,364	1,703,349	1,644,015	4,825,020	2,660,141	2,164,879
	Height (cm)	153.63 ± 8.22	152.94 ± 8.79	154.35 ± 7.51	155.69 ± 8.56	156.4 ± 8.15	154.84 ± 8.97
	Weight (kg)	46.3 ± 10.55	45.18 ± 10.31	47.46 ± 10.66	54.05 ± 15.3	53.45 ± 13.0	54.78 ± 17.65
	BMI	19.49 ± 3.49	19.18 ± 3.32	19.81 ± 3.64	22.05 ± 4.94	21.67 ± 4.38	22.5 ± 5.53
8	*N*	3,617,264	1,867,912	1,749,352	3,969,663	1,856,103	2,113,560
	Height (cm)	159.53 ± 8.29	160.66 ± 9.25	158.33 ± 6.93	162.21 ± 7.95	164.37 ± 7.7	160.29 ± 7.67
	Weight (kg)	51.36 ± 10.77	51.28 ± 10.98	51.43 ± 10.55	61.36 ± 18.05	63.32 ± 17.69	59.67 ± 18.13
	BMI	20.06 ± 3.33	19.71 ± 3.04	20.44 ± 3.57	23.28 ± 6.44	23.47 ± 6.44	23.11 ± 6.44
9	*N*	3,668,706	1,871,570	1,797,136	4,382,750	2,009,846	2,372,903
	Height (cm)	164.05 ± 8.15	166.73 ± 8.69	161.26 ± 6.47	165.15 ± 7.14	168.89 ± 6.29	161.99 ± 6.23
	Weight (kg)	56.41 ± 11.25	58.3 ± 12.8	54.44 ± 8.95	62.15 ± 16.49	63.68 ± 16.67	60.84 ± 16.22
	BMI	20.87 ± 3.40	20.83 ± 3.62	20.92 ± 3.15	22.64 ± 5.14	22.18 ± 5.02	23.04 ±5.21
10	*N*	3,730,948	1,958,882	1,772,066	3,289,875	1,961,082	1,328,794
	Height (cm)	167.43 ± 8.89	171.97 ± 8.06	162.42 ± 6.84	168.94 ± 9.50	174.03 ± 7.82	161.43 ± 6.21
	Weight (kg)	60.76 ± 12.01	63.58 ± 12.37	57.64 ± 10.77	67.99 ± 17.21	71.79 ± 16.71	62.25 ± 16.32
	BMI	21.6 ± 3.49	21.42 ± 3.45	21.8 ± 3.54	23.60 ± 5.09	23.60 ± 4.65	23.84 ± 5.67
Total	*N*	19,043,317	9,744,373	9,298,944	24,612,602	12,360,025	12,252,577
	Height (cm)	157.81 ± 11.29	159.27 ± 12.8	156.29 ± 9.20	157.32 ± 11.73	159.37 ± 12.97	155.25 ± 9.92
	Weight (kg)	50.65 ± 13.1	51.32 ± 14.20	49.98 ± 11.78	55.4 ± 18.04	57.1 ± 18.49	53.71 ± 17.45
	BMI	20.08 ± 3.57	19.89 ± 3.5	20.28 ± 3.62	22 ± 5.42	22.07 ± 5.32	21.92 ± 5.51

**Table 2:** Ranking of physical activities in school physical education (PE) in 1985 (Body mass index (BMI): 1 = Underweight, 2 = Normal weight, 3 = Overweight, 4 = Obese).

Activity	Total						Boys						Girls					
	All		BMI				All		BMI				All		BMI			
	%	Rank	1	2	3	4	%	Rank	1	2	3	4	%	Rank	1	2	3	4
Basketball	83.60	1	90.30%	83.40%	84.40%	81.20%	84.20	1	92.30%	84.30%	84.20%	81.30%	82.90	1	88.10%	82.50%	84.60%	81.10%
Jogging (distance running)	73.40	2	75.50%	73.30%	74.70%	70.80%	73.10	3	75.50%	73.00%	73.50%	72.30%	73.60	3	75.60%	73.50%	75.80%	68.90%
Baseball/softball	71.50	3	74.00%	70.70%	74.90%	72.50%	70.80	4	72.30%	70.00%	73.30%	73.60%	72.30	5	75.90%	71.40%	76.50%	71.30%
Calisthenics/exercises	71.40	4	74.00%	71.40%	71.40%	71.10%	70.60	5	72.90%	70.90%	67.70%	72.20%	72.30	4	75.10%	71.90%	75.00%	69.80%
Volleyball	71.30	5	78.20%	71.60%	70.90%	66.60%	66.30	6	75.40%	67.00%	61.20%	65.80%	76.60	2	81.20%	76.40%	80.40%	67.60%
Play games	66.90	6	76.40%	66.60%	67.50%	65.00%	74.10	2	85.10%	73.30%	76.80%	72.20%	59.40	9	67.40%	59.60%	58.50%	56.50%
Soccer	63.80	7	72.90%	64.00%	63.50%	59.20%	65.20	7	71.70%	65.30%	65.90%	60.40%	62.40	7	74.10%	62.60%	61.20%	57.70%
Relays	59.20	8	59.50%	59.40%	58.00%	59.60%	56.30	11	57.70%	57.10%	51.70%	56.40%	62.20	8	61.30%	61.80%	64.20%	63.30%
Kickball	58.60	9	67.80%	58.20%	57.70%	61.00%	54.80	13	58.80%	55.10%	50.50%	58.60%	62.60	6	77.20%	61.60%	64.60%	63.80%
Football	56.40	10	55.20%	56.10%	58.00%	57.00%	57.40	9	56.90%	57.00%	59.10%	58.50%	55.30	10	53.40%	55.10%	56.90%	55.10%
Backyard games	56.20	11	50.80%	56.40%	55.40%	57.10%	62.40	8	57.20%	62.80%	60.60%	63.10%	49.70	14	44.10%	49.70%	50.40%	50.00%
Running sprints	54.90	12	55.10%	55.70%	53.80%	48.80%	56.70	10	62.40%	57.90%	52.30%	51.80%	53.00	11	47.40%	53.40%	55.20%	45.20%
Gymnastics	53.70	13	53.60%	53.40%	54.10%	55.40%	55.40	12	56.00%	56.00%	52.60%	54.80%	51.90	12	51.20%	50.70%	55.70%	56.10%
Dance	44.60	14	47.80%	44.40%	44.80%	44.70%	43.90	14	51.00%	43.90%	41.70%	45.30%	45.20	15	44.50%	44.90%	47.90%	43.90%
Jumping rope	43.80	15	52.50%	43.70%	42.80%	44.40%	37.20	15	46.90%	37.10%	34.90%	38.30%	50.80	13	58.30%	50.60%	50.50%	51.50%
Track and field	29.60	16	29.10%	29.50%	30.90%	28.10%	29.60	16	28.80%	29.20%	31.70%	29.70%	29.60	16	29.40%	29.70%	30.20%	26.20%
Field hockey	27.30	17	29.40%	27.70%	26.80%	23.10%	28.80	17	34.90%	29.40%	27.40%	23.70%	25.70	17	23.70%	26.00%	26.20%	22.50%
Stickball	21.50	18	17.30%	20.30%	24.30%	30.10%	22.80	18	15.20%	21.70%	23.90%	35.00%	20.10	19	19.40%	18.90%	24.70%	24.20%
Badminton	20.20	19	16.20%	20.10%	20.40%	22.30%	17.70	21	8.50%	17.90%	17.20%	20.70%	22.80	18	24.10%	22.40%	23.50%	24.20%
Weightlifting or training	19.40	20	18.50%	19.70%	19.80%	15.60%	21.80	19	15.00%	21.80%	24.90%	18.60%	16.80	22	22.10%	17.50%	14.90%	12.20%
Walking quickly	17.30	21	14.90%	17.00%	17.10%	22.30%	15.30	22	14.90%	14.80%	14.90%	21.00%	19.50	20	15.00%	19.30%	19.20%	23.80%
Tennis	16.10	22	22.70%	16.20%	15.30%	14.50%	14.00	23	23.30%	14.20%	12.90%	11.50%	18.30	21	22.00%	18.40%	17.80%	18.00%
Ping-pong (table tennis)	13.70	23	16.60%	13.50%	12.90%	16.90%	12.80	25	14.70%	12.50%	11.50%	17.50%	14.70	23	18.40%	14.50%	14.30%	16.20%
Bowling/duckpins	13.00	24	8.10%	12.90%	12.80%	15.20%	12.40	26	7.70%	12.20%	11.90%	17.20%	13.50	24	8.60%	13.70%	13.70%	12.90%
Wrestling	12.60	25	11.30%	12.50%	13.70%	12.00%	21.40	20	19.40%	21.10%	24.40%	20.10%	3.40	32	2.90%	3.50%	3.30%	2.50%
Swimming/diving	12.60	26	19.60%	12.50%	14.70%	7.00%	12.80	24	17.20%	12.60%	15.30%	8.20%	12.40	25	22.20%	12.30%	14.20%	5.60%
Frisbee	10.70	27	10.10%	10.40%	10.70%	14.00%	9.60	27	7.40%	9.50%	8.50%	13.20%	11.80	26	13.00%	11.30%	12.80%	14.90%
Handball	9.80	28	4.50%	9.80%	11.10%	9.80%	9.50	28	6.40%	9.70%	10.20%	7.60%	10.20	27	2.60%	9.90%	11.90%	12.30%
Archery	7.60	29	13.20%	7.20%	8.80%	7.20%	7.80	29	13.30%	7.20%	10.10%	7.00%	7.40	29	13.10%	7.10%	7.50%	7.50%
Bicycling	4.90	30	5.70%	4.70%	5.40%	5.40%	4.80	30	5.70%	4.70%	5.00%	5.80%	5.00	31	5.80%	4.80%	5.80%	4.90%
Cheerleading	4.40	31	5.10%	4.20%	4.70%	5.70%	1.30	44	2.20%	1.30%	0.40%	2.90%	7.70	28	8.10%	7.30%	9.00%	9.00%
Roller skating	4.40	32	4.20%	4.20%	4.70%	5.60%	3.60	32	0.70%	3.60%	3.70%	4.40%	5.20	30	7.80%	4.90%	5.80%	7.00%
Water polo	3.50	33	7.10%	3.60%	2.80%	2.40%	4.30	31	9.20%	4.30%	3.50%	3.30%	2.60	37	4.90%	2.80%	2.00%	1.30%
Golf	3.00	34	1.00%	2.90%	3.30%	3.70%	3.20	33	0.80%	3.10%	3.40%	5.30%	2.70	36	1.30%	2.70%	3.20%	1.80%
Horseshoes	2.70	35	3.30%	2.60%	2.40%	3.50%	2.90	34	0.30%	2.90%	2.70%	5.20%	2.30	38	6.40%	2.30%	2.10%	1.60%
Racquetball	2.50	36	2.40%	2.60%	2.50%	1.60%	2.20	37	1.90%	2.40%	2.10%	0.90%	2.80	34	2.80%	2.90%	2.90%	2.40%
Karate/judo/martial arts	2.50	37	5.60%	2.40%	2.60%	2.20%	2.20	38	5.70%	2.00%	3.10%	1.10%	2.80	35	5.60%	2.80%	2.20%	3.50%
Marching/drills/band	2.20	38	1.30%	2.10%	3.20%	2.30%	1.60	42	0.90%	1.50%	2.40%	0.90%	2.90	33	1.70%	2.60%	3.90%	4.10%
Lacrosse	2.10	39	1.70%	2.10%	1.70%	2.20%	2.60	35	2.50%	2.60%	2.00%	3.30%	1.50	42	0.80%	1.60%	1.40%	0.80%
Rugby	1.90	40	0.70%	1.80%	2.40%	2.10%	1.90	41	0.00%	1.90%	2.40%	1.70%	1.80	39	1.50%	1.70%	2.40%	2.60%
Hiking/backpacking	1.80	41	0.60%	1.90%	1.50%	2.40%	2.10	39	0.00%	2.10%	1.90%	3.60%	1.50	41	1.30%	1.70%	1.20%	0.90%
Boxing	1.60	42	1.90%	1.60%	1.50%	2.00%	2.50	36	3.00%	2.50%	2.30%	3.30%	0.70	50	0.70%	0.70%	0.80%	0.50%
Fishing	1.50	43	0.00%	1.40%	2.40%	2.10%	2.10	40	0.00%	1.80%	3.10%	3.20%	1.00	46	0.00%	0.90%	1.70%	0.70%
Canoeing/kayaking	1.20	44	1.90%	1.10%	1.40%	1.60%	0.80	49	0.00%	0.70%	1.20%	1.40%	1.60	40	3.90%	1.60%	1.50%	2.00%
Fencing	1.10	45	1.30%	1.10%	1.30%	0.40%	1.30	45	1.50%	1.30%	1.60%	0.80%	0.90	48	1.00%	1.00%	0.90%	0.00%
Riflery/shooting sports	1.10	46	1.80%	1.00%	1.30%	1.30%	1.50	43	3.60%	1.30%	2.00%	1.50%	0.60	51	0.00%	0.60%	0.70%	1.20%
Yoga	1.00	47	1.90%	1.00%	1.00%	0.80%	0.50	55	3.10%	0.50%	0.20%	0.00%	1.50	43	0.70%	1.50%	1.70%	1.70%
Rock climbing	1.00	48	0.60%	1.00%	1.10%	1.00%	1.00	46	0.00%	1.00%	1.40%	1.60%	0.90	47	1.30%	1.00%	0.80%	0.30%
Skateboarding	0.90	49	0.80%	1.00%	0.60%	0.90%	0.70	50	0.70%	0.80%	0.40%	1.00%	1.10	44	0.80%	1.20%	0.90%	0.90%
Ice skating	0.90	50	0.50%	1.00%	0.60%	0.00%	0.70	51	0.00%	0.90%	0.40%	0.00%	1.00	45	1.10%	1.10%	0.80%	0.00%
Sledding	0.70	51	0.40%	0.80%	0.40%	1.10%	0.90	47	0.80%	1.00%	0.70%	0.80%	0.60	53	0.00%	0.60%	0.20%	1.50%
Horseback riding	0.70	52	0.50%	0.70%	1.20%	0.20%	0.50	53	0.00%	0.50%	1.10%	0.00%	0.90	49	1.10%	0.90%	1.30%	0.50%
Skiing (cross country)	0.60	53	0.00%	0.60%	0.70%	0.00%	0.50	54	0.00%	0.50%	0.90%	0.00%	0.60	52	0.00%	0.70%	0.50%	0.00%
Waterskiing	0.50	54	0.80%	0.60%	0.40%	0.20%	0.80	48	1.50%	1.00%	0.30%	0.00%	0.30	57	0.00%	0.20%	0.50%	0.50%
Skiing (downhill)	0.40	55	0.00%	0.40%	0.50%	0.70%	0.60	52	0.00%	0.60%	0.70%	1.00%	0.20	58	0.00%	0.20%	0.40%	0.40%
Sailing	0.40	56	0.00%	0.40%	0.30%	1.00%	0.40	58	0.00%	0.40%	0.10%	1.10%	0.40	54	0.00%	0.40%	0.40%	0.90%
Hunting	0.40	57	0.90%	0.30%	0.70%	0.50%	0.40	57	0.00%	0.40%	0.70%	0.40%	0.30	55	1.90%	0.20%	0.70%	0.50%
Ice hockey	0.30	58	0.50%	0.30%	0.60%	0.20%	0.40	56	0.00%	0.40%	0.80%	0.00%	0.20	59	1.10%	0.10%	0.30%	0.50%
Surfing	0.30	59	0.00%	0.20%	0.40%	0.20%	0.20	59	0.00%	0.30%	0.00%	0.00%	0.30	56	0.00%	0.20%	0.80%	0.50%
Rowing/crew	0.10	60	0.50%	0.10%	0.40%	0.00%	0.10	61	0.00%	0.10%	0.20%	0.00%	0.20	60	1.00%	0.10%	0.50%	0.00%
Hang gliding	0.10	61	0.00%	0.10%	0.30%	0.20%	0.10	60	0.00%	0.10%	0.00%	0.40%	0.20	61	0.00%	0.10%	0.60%	0.00%

**Table 3:** Ranking of physical activities out of school PE in 1985 (BMI: 1 = Underweight, 2 = Normal weight, 3 = Overweight, 4 = Obese).

Activity	Total						Boys						Girls					
	All		BMI				All		BMI				All	BMI				
	%	Rank	1	2	3	4	%	Rank	1	2	3	4	%	Rank	1	2	3	4
Basketball	76.90	3	75.90%	77.40%	75.60%	75.10%	85.10	1	92.80%	85.50%	84.40%	80.20%	68.30	8	58.30%	68.90%	67.00%	69.00%
Jogging (distance running)	61.50	9	62.00%	63.50%	56.30%	50.10%	62.20	9	65.90%	64.00%	57.60%	51.70%	60.70	10	58.00%	63.10%	55.10%	48.30%
Baseball/softball	74.40	5	76.10%	74.30%	75.60%	72.60%	80.10	4	79.30%	80.10%	82.20%	76.00%	68.50	6	72.90%	68.30%	69.10%	68.70%
Calisthenics/exercises	42.60	20	43.50%	43.30%	40.80%	38.40%	37.60	24	43.50%	37.70%	37.30%	34.80%	47.70	15	43.50%	49.10%	44.10%	42.60%
Volleyball	54.70	14	50.80%	54.40%	58.70%	51.90%	46.60	17	41.50%	46.20%	50.70%	43.70%	63.30	9	60.50%	62.90%	66.50%	61.40%
Play games	75.20	4	75.00%	75.70%	72.60%	75.50%	75.40	5	73.10%	76.30%	73.50%	71.50%	75.00	4	77.10%	75.10%	71.80%	80.20%
Soccer	53.00	15	57.70%	53.60%	51.30%	47.80%	60.20	10	67.30%	61.00%	58.20%	53.80%	45.30	19	47.80%	45.80%	44.50%	40.80%
Relays	43.30	19	46.30%	44.40%	39.50%	37.50%	39.10	23	42.20%	40.70%	34.80%	29.50%	47.60	16	50.50%	48.30%	44.00%	46.90%
Kickball	55.40	13	64.60%	54.70%	56.10%	58.90%	54.10	14	59.80%	53.90%	54.10%	53.80%	56.80	12	69.50%	55.40%	58.00%	64.80%
Football	70.70	6	76.20%	70.80%	69.80%	68.90%	71.80	6	79.20%	72.00%	70.60%	69.70%	69.50	5	73.00%	69.60%	69.00%	68.00%
Backyard games	68.50	8	71.20%	69.00%	65.50%	67.80%	68.40	8	66.00%	69.20%	66.50%	65.10%	68.50	7	76.60%	68.80%	64.60%	71.00%
Running sprints	39.70	22	38.30%	41.90%	32.70%	31.60%	46.20	18	47.70%	48.90%	37.50%	34.60%	33.00	25	28.50%	34.60%	28.10%	28.10%
Gymnastics	45.00	17	44.20%	44.90%	46.10%	44.40%	45.00	19	48.20%	45.40%	44.10%	41.30%	45.00	20	39.90%	44.30%	48.10%	48.10%
Dance	55.90	12	47.90%	56.60%	55.80%	51.50%	55.40	13	53.00%	56.20%	55.90%	47.20%	56.50	14	42.60%	57.10%	55.70%	56.70%
Jumping rope	44.80	18	50.20%	44.40%	45.60%	44.70%	32.40	28	37.00%	32.60%	31.80%	29.30%	57.70	11	64.10%	56.80%	59.10%	62.80%
Track and field	19.20	37	19.60%	20.00%	18.00%	12.90%	21.10	40	24.60%	22.00%	19.50%	14.40%	17.20	34	14.50%	18.00%	16.40%	11.10%
Field hockey	17.90	41	17.80%	18.10%	17.30%	16.80%	25.20	37	24.60%	25.60%	24.40%	23.60%	10.10	46	10.80%	10.10%	10.40%	8.90%
Stickball	22.50	34	21.00%	22.20%	22.70%	25.10%	30.80	30	26.00%	30.80%	29.90%	33.70%	13.80	40	15.70%	13.20%	15.80%	14.90%
Badminton	22.80	32	20.30%	23.10%	21.30%	23.00%	22.60	39	19.10%	23.00%	20.50%	23.80%	22.90	30	21.60%	23.20%	22.10%	22.00%
Weightlifting or training	32.70	26	26.80%	32.90%	34.00%	30.20%	46.60	16	42.00%	45.90%	52.60%	43.90%	18.10	33	10.90%	19.20%	15.90%	14.10%
Walking quickly	37.30	24	44.70%	37.40%	36.30%	36.00%	29.50	31	37.50%	29.40%	29.10%	28.00%	45.50	18	52.10%	45.80%	43.30%	45.50%
Tennis	40.40	21	47.80%	41.00%	39.90%	32.10%	36.70	26	44.00%	37.40%	36.00%	28.10%	44.20	21	51.90%	44.80%	43.60%	36.70%
Ping-pong (table tennis)	46.90	16	56.10%	47.50%	43.40%	44.30%	47.50	15	64.00%	48.10%	43.80%	43.30%	46.30	17	48.00%	46.90%	43.10%	45.40%
Bowling/duckpins	37.60	23	30.30%	37.70%	39.00%	36.40%	39.60	22	36.80%	39.50%	41.00%	38.90%	35.60	24	23.50%	35.90%	37.00%	33.50%
Wrestling	27.80	29	26.20%	27.00%	29.80%	32.60%	41.60	20	32.50%	41.50%	41.90%	45.80%	13.30	41	19.50%	11.80%	18.00%	17.00%
Swimming/diving	85.70	1	84.90%	86.20%	86.10%	80.50%	83.80	2	86.00%	84.70%	82.70%	76.40%	87.70	1	83.70%	87.70%	89.40%	85.40%
Frisbee	57.70	10	59.80%	57.60%	58.20%	56.70%	58.70	12	63.40%	59.30%	56.20%	55.70%	56.60	13	56.00%	55.80%	60.20%	58.00%
Handball	21.80	36	21.00%	21.20%	25.30%	20.70%	24.00	38	16.50%	24.40%	27.00%	17.00%	19.50	32	25.70%	17.90%	23.60%	25.00%
Archery	18.60	38	19.60%	18.20%	19.30%	20.90%	28.40	33	32.40%	27.30%	31.90%	31.40%	8.30	48	6.20%	8.60%	7.00%	8.60%
Bicycling	82.80	2	87.80%	82.90%	82.30%	80.40%	82.30	3	90.90%	83.00%	79.20%	78.20%	83.30	2	84.60%	82.90%	85.30%	82.90%
Cheerleading	12.00	48	12.60%	12.40%	11.10%	10.00%	0.40	61	0.00%	0.30%	1.10%	0.30%	24.20	29	25.80%	25.10%	20.90%	21.40%
Roller skating	69.20	7	68.50%	69.70%	71.20%	59.60%	60.20	11	58.20%	61.20%	61.40%	47.90%	78.60	3	79.10%	78.60%	80.80%	73.40%
Water polo	9.90	52	11.50%	9.80%	11.20%	7.40%	10.70	50	14.60%	11.00%	10.20%	7.20%	9.00	47	8.30%	8.50%	12.10%	7.70%
Golf	18.50	39	23.80%	19.00%	16.60%	15.50%	25.40	36	30.20%	26.10%	23.90%	20.40%	11.20	44	17.00%	11.50%	9.40%	9.60%
Horseshoes	22.80	33	20.30%	22.80%	21.10%	28.00%	28.90	32	25.90%	28.70%	27.30%	34.80%	16.50	35	14.40%	16.50%	15.10%	19.90%
Racquetball	17.50	43	16.70%	18.00%	16.90%	14.40%	19.90	43	23.00%	20.20%	20.20%	15.40%	15.10	37	10.10%	15.70%	13.70%	13.30%
Karate/judo/martial arts	11.70	49	11.80%	12.00%	10.90%	9.40%	17.80	47	18.90%	18.50%	17.00%	12.40%	5.30	50	4.40%	5.30%	5.00%	6.00%
Marching/drills/band	11.70	50	12.10%	11.90%	10.30%	11.60%	9.30	52	12.70%	9.50%	8.00%	8.50%	14.10	39	11.50%	14.40%	12.50%	15.40%
Lacrosse	2.40	59	2.40%	2.60%	2.00%	1.20%	3.40	57	4.00%	3.70%	2.80%	1.90%	1.30	58	0.80%	1.40%	1.30%	0.40%
Rugby	5.20	56	6.10%	5.00%	6.10%	4.80%	9.00	53	12.10%	8.80%	10.80%	6.70%	1.10	59	0.00%	0.90%	1.70%	2.50%
Hiking/backpacking	24.50	31	26.00%	25.60%	21.00%	20.40%	27.70	35	28.60%	28.40%	25.90%	23.90%	21.20	31	23.30%	22.60%	16.20%	16.20%
Boxing	10.70	51	11.00%	10.90%	11.10%	7.90%	18.00	46	18.80%	18.70%	18.10%	10.90%	3.00	56	2.90%	2.60%	4.30%	4.30%
Fishing	56.50	11	59.40%	55.40%	61.60%	56.80%	69.00	7	73.90%	67.90%	73.30%	70.10%	43.50	22	44.40%	42.30%	50.20%	41.20%
Canoeing/kayaking	17.50	44	20.20%	18.10%	15.10%	14.60%	20.50	42	25.80%	21.50%	17.40%	13.90%	14.40	38	14.30%	14.60%	12.70%	15.50%
Fencing	2.20	60	2.70%	2.10%	2.00%	2.80%	3.20	58	2.50%	3.30%	2.40%	4.50%	1.10	60	3.00%	0.90%	1.60%	0.80%
Riflery/shooting sports	18.50	40	20.90%	18.50%	17.70%	18.90%	31.30	29	34.90%	31.10%	32.70%	29.60%	5.00	51	6.30%	5.20%	3.10%	6.40%
Yoga	3.40	58	4.10%	3.60%	2.10%	3.20%	2.40	59	4.00%	2.50%	1.30%	2.20%	4.50	52	4.20%	4.80%	2.80%	4.50%
Rock climbing	14.80	45	14.80%	15.10%	12.80%	15.10%	18.60	45	20.00%	19.30%	15.30%	18.00%	10.70	45	9.50%	10.80%	10.30%	11.70%
Skateboarding	35.40	25	38.40%	36.30%	33.20%	30.30%	41.50	21	45.70%	43.30%	35.90%	32.70%	29.10	27	30.90%	28.90%	30.60%	27.40%
Ice skating	25.50	30	20.70%	26.50%	23.80%	20.60%	20.90	41	18.70%	21.60%	20.60%	15.90%	30.30	26	22.80%	31.70%	26.90%	26.10%
Sledding	31.80	27	26.80%	31.80%	32.40%	32.30%	35.30	27	33.50%	34.80%	38.20%	35.60%	28.20	28	19.80%	28.80%	26.80%	28.40%
Horseback riding	31.80	28	28.50%	32.10%	32.50%	27.90%	27.80	34	27.60%	28.30%	28.60%	22.30%	35.90	23	29.40%	36.20%	36.30%	34.40%
Skiing (cross country)	4.70	57	2.80%	4.80%	5.30%	3.40%	5.20	56	2.60%	5.10%	7.30%	2.90%	4.10	54	2.90%	4.40%	3.30%	4.00%
Waterskiing	17.80	42	14.60%	18.70%	17.80%	9.50%	19.90	44	20.60%	21.10%	18.60%	9.30%	15.60	36	8.40%	16.10%	17.10%	9.90%
Skiing (downhill)	14.10	46	11.90%	14.70%	14.30%	7.50%	15.30	48	16.70%	15.90%	15.50%	8.50%	12.80	42	6.90%	13.50%	13.20%	6.30%
Sailing	12.80	47	14.30%	13.40%	11.90%	8.10%	13.50	49	19.60%	14.00%	12.10%	8.70%	12.10	43	8.80%	12.70%	11.80%	7.30%
Hunting	22.10	35	19.00%	22.20%	22.20%	22.40%	37.60	25	34.30%	37.90%	37.40%	35.70%	6.00	49	3.00%	5.70%	7.40%	6.80%
Ice hockey	5.90	54	5.60%	6.00%	6.10%	5.10%	10.20	51	8.30%	10.40%	9.80%	9.50%	1.50	57	2.80%	1.40%	2.60%	0.00%
Surfing	5.90	55	7.10%	6.40%	5.10%	2.80%	8.10	54	9.40%	8.80%	6.80%	3.00%	3.70	55	4.60%	3.80%	3.50%	2.60%
Rowing/crew	6.10	53	7.50%	6.10%	6.10%	6.20%	7.80	55	7.80%	7.70%	7.80%	8.90%	4.40	53	7.10%	4.40%	4.50%	3.10%
Hang gliding	0.90	61	0.40%	0.80%	1.20%	1.40%	1.10	60	0.80%	1.10%	1.50%	1.30%	0.70	61	0.00%	0.60%	1.00%	1.40%

**Table 4:** The correlation between inside and outside PE ranking (BMI: 1 = Underweight, 2 = Normal weight, 3 = Overweight, 4 = Obese).

		BMI Category									
1985		Boys					Girls				
Grade	Total	1	2	3	4	Total	1	2	3	4	Total
5	0.727	0.627	0.688	0.400	0.596	0.685	0.407	0.709	0.573	0.531	0.698
6	0.718	0.482	0.704	0.699	0.529	0.713	0.536	0.785	0.656	0.529	0.753
7	0.684	0.397	0.706	0.538	0.548	0.719	0.508	0.711	0.718	0.689	0.755
8	0.676	0.584	0.669	0.588	0.588	0.672	0.612	0.636	0.648	0.458	0.699
9	0.653	0.583	0.666	0.628	0.594	0.675	0.305	0.639	0.609	0.498	0.646
10	0.691	0.726	0.581	0.649	0.390	0.595	0.582	0.639	0.606	0.582	0.649
Total	0.702	0.609	0.711	0.702	0.642		0.685	0.728	0.700	0.697	
**2012**											
**Grade**	**Total**	**1**	**2**	**3**	**4**	**Total**	**1**	**2**	**3**	**4**	**Total**
5	0.497	-	0.300	−0.400	0.200	0.500	-	−0.683	-	−0.866	0.006
6	0.681	-	0.833	−0.500	0.500	0.718	-	0.606	−0.500	−0.203	0.533
7	0.714	-	0.794	0.257	0.771	0.664	-	0.595	0.300	−0.300	0.655
8	0.213	-	0.167	−0.205	0.429	0.364	-	−0.150	−1.000 ^a^	-	0.091
9	0.503	-	0.429	-	1.000 ^a^	0.794	-	0.064	-	0.500	−0.011
10	0.483	-	0.350	−1.000 ^a^	−1.000 ^a^	0.308	-	−0.200	0.500	−1.000 ^a^	0.188
Total	0.556	-	0.652	0.467	0.617		-	0.362	0.248	0.484	

“-” Can not be computed because the sample is 0 or 1; ^a^ The compare sample less than three variables.

**Table 5:** Ranking of physical activities (PA) inside school in 2012 (BMI: 1 = Underweight, 2 = Normal weight, 3 = Overweight, 4 = Obese); NA = No participation by boys or girls in this specific activity.

Activity	Total						Boys						Girls					
	All		BMI				All		BMI				All		BMI			
	%	Rank	1	2	3	4	%	Rank	1	2	3	4	%	Rank	1	2	3	4
Basketball	18.00	1	7.00%	19.70%	17.10%	15.80%	21.90	1	12.00%	22.90%	25.00%	18.80%	14.00	1	0.00%	16.80%	10.20%	11.30%
Soccer	12.00	2	2.00%	13.10%	14.50%	7.60%	13.90	4	3.40%	15.90%	18.60%	6.60%	10.10	2	0.00%	10.70%	10.80%	8.90%
Baseball	10.10	3	0.00%	10.00%	15.60%	6.10%	14.10	3	0.00%	14.90%	23.00%	6.90%	6.20	4	0.00%	5.60%	9.10%	5.00%
Football	9.30	4	0.00%	9.60%	8.00%	11.50%	17.90	2	0.00%	19.90%	17.00%	16.70%	0.80	15	0.00%	0.30%	0.00%	3.70%
Track and field	7.00	5	0.00%	9.00%	4.70%	4.80%	8.00	5	0.00%	9.10%	7.10%	7.40%	6.10	5	0.00%	9.00%	2.60%	0.80%
Volleyball	5.60	6	0.00%	6.60%	8.50%	0.70%	2.40	9	0.00%	3.30%	3.50%	0.00%	8.80	3	0.00%	9.50%	13.00%	1.70%
Running	4.20	7	7.00%	4.90%	4.00%	1.80%	4.60	6	12.00%	4.20%	8.40%	1.30%	3.70	8	0.00%	5.60%	0.00%	2.40%
Dance	3.20	8	10.20%	3.20%	1.50%	3.80%	1.10	14	0.00%	2.00%	0.00%	0.00%	5.30	7	24.50%	4.30%	2.80%	9.50%
Cheerleading	2.90	9	0.00%	3.00%	2.20%	3.70%	NA						5.80	6	0.00%	5.80%	4.20%	9.20%
Swimming/diving	1.80	10	0.00%	1.80%	1.90%	1.70%	1.90	10	0.00%	2.90%	0.00%	1.40%	1.70	11	0.00%	0.90%	3.60%	2.10%
Tennis	1.70	11	0.00%	1.70%	2.10%	1.40%	1.00	15	0.00%	1.40%	0.00%	1.00%	2.30	9	0.00%	1.90%	4.00%	1.90%
Golf	1.60	12	0.00%	2.40%	0.80%	0.60%	2.90	8	0.00%	4.30%	1.60%	1.00%	0.40	16	0.00%	0.70%	0.00%	0.00%
Gymnastics	1.60	12	0.00%	1.80%	1.50%	1.40%	0.90	16	0.00%	1.70%	0.00%	0.00%	2.20	10	0.00%	1.80%	2.90%	3.50%
Wrestling	1.60	12	0.00%	2.00%	0.00%	2.70%	3.30	7	0.00%	4.30%	0.00%	4.40%	NA					
Lacrosse	1.40	15	4.00%	2.00%	0.80%	0.00%	1.50	12	0.00%	2.20%	1.60%	0.00%	1.30	12	9.60%	1.80%	0.00%	0.00%
Field hockey	1.20	16	0.00%	0.50%	3.40%	0.70%	1.60	11	0.00%	0.40%	6.70%	0.00%	0.80	14	0.00%	0.60%	0.50%	1.70%
Trampoline	0.60	17	0.00%	1.10%	0.00%	0.00%	1.30	13	0.00%	2.40%	0.00%	0.00%	NA					
Martial arts	0.40	18	0.00%	0.70%	0.00%	0.00%	NA						0.80	13	0.00%	1.40%	0.00%	0.00%
Frisbee	0.30	19	0.00%	0.50%	0.00%	0.00%	0.60	17	0.00%	1.10%	0.00%	0.00%	NA					
Walking quickly	0.20	20	0.00%	0.20%	0.60%	0.00%	0.30	18	0.00%	0.00%	1.30%	0.00%	0.20	17	0.00%	0.30%	0.00%	0.00%
Bocce ball	0.10	21	0.00%	0.20%	0.00%	0.00%	0.20	19	0.00%	0.40%	0.00%	0.00%	NA					

**Table 6:** Ranking of PA outside school in 2012 (BMI: 1 = Underweight, 2 = Normal weight, 3 = Overweight, 4 = Obese); NA = No participation by boys or girls in this specific activity.

Activity	Total						Boys						Girls					
	All		BMI				All		BMI				All		BMI			
	%	Rank	1	2	3	4	%	Rank	1	2	3	4	%	Rank	1	2	3	4
Running	26.90	1	14.80%	29.20%	25.50%	23.50%	29.70	2	15.10%	28.90%	35.90%	28.40%	24.10	1	14.30%	29.50%	16.20%	16.30%
Basketball	26.40	2	26.30%	26.50%	28.60%	23.60%	37.90	1	38.40%	40.70%	41.40%	28.00%	14.90	5	9.50%	13.80%	17.30%	16.90%
Walking	22.60	3	14.80%	21.30%	22.80%	27.50%	22.50	5	18.50%	20.10%	28.50%	23.70%	22.60	2	9.60%	22.30%	17.80%	33.30%
Bicycling	21.60	4	18.80%	20.60%	22.80%	23.60%	27.30	3	25.30%	26.50%	31.00%	26.30%	15.80	4	9.60%	15.30%	15.60%	19.40%
Football	12.60	5	16.90%	11.00%	11.90%	17.50%	22.50	4	29.10%	21.50%	22.20%	24.20%	2.70	19	0.00%	1.60%	2.60%	7.60%
Soccer	12.50	6	15.00%	12.70%	12.60%	11.70%	15.70	7	0.00%	16.80%	17.40%	14.30%	9.40	7	35.90%	9.00%	8.20%	8.00%
Backyard games	12.50	6	15.40%	12.80%	12.10%	11.20%	13.20	8	12.20%	13.20%	17.10%	9.90%	11.80	6	19.90%	12.50%	7.70%	13.20%
Baseball	11.40	8	0.00%	10.90%	17.00%	8.50%	16.80	6	0.00%	17.50%	25.50%	10.10%	6.00	12	0.00%	5.00%	9.40%	6.20%
Dance	10.60	9	15.00%	10.10%	12.90%	8.90%	3.50	18	0.00%	3.50%	4.30%	3.20%	17.80	3	35.90%	16.10%	20.60%	17.30%
Play games	9.50	10	7.00%	9.90%	11.90%	6.30%	10.60	9	12.00%	10.90%	14.30%	6.20%	8.50	9	0.00%	8.90%	9.70%	6.40%
Swimming/diving	8.30	11	4.00%	8.90%	8.60%	7.10%	7.40	12	0.00%	8.40%	9.00%	4.70%	9.30	8	9.60%	9.30%	8.20%	10.60%
Aerobics	7.20	12	4.40%	6.80%	4.20%	12.10%	8.90	10	7.60%	8.40%	5.90%	12.80%	5.50	13	0.00%	5.30%	2.60%	11.00%
Volleyball	4.40	13	0.00%	4.80%	2.40%	5.90%	1.40	27	0.00%	1.80%	0.00%	1.70%	7.40	10	0.00%	7.50%	4.50%	12.20%
Skateboarding	4.30	14	7.10%	4.30%	3.90%	4.50%	7.40	11	12.20%	6.80%	8.20%	7.50%	1.20	25	0.00%	2.10%	0.00%	0.00%
Jumping rope	4.20	15	0.00%	4.80%	3.90%	3.40%	2.20	25	0.00%	2.40%	0.00%	3.80%	6.20	11	0.00%	6.90%	7.40%	2.80%
Trampoline	4.10	16	0.00%	4.40%	3.70%	4.40%	4.80	13	0.00%	3.40%	6.60%	7.40%	3.40	16	0.00%	5.30%	1.20%	0.00%
Tennis	3.50	17	0.00%	3.70%	4.50%	2.20%	3.80	17	0.00%	4.20%	6.00%	1.40%	3.20	17	0.00%	3.30%	3.10%	3.40%
Scooter riding	3.30	18	7.10%	3.20%	3.20%	2.90%	4.20	15	12.20%	4.80%	2.60%	2.90%	2.30	21	0.00%	1.90%	3.70%	2.80%
Hiking	3.20	19	6.50%	3.50%	0.90%	4.50%	4.10	16	11.10%	3.50%	0.50%	7.50%	2.40	20	0.00%	3.60%	1.20%	0.00%
Track and field	3.00	20	0.00%	4.80%	0.90%	0.60%	3.20	20	0.00%	5.30%	1.90%	0.00%	3.90	18	0.00%	4.70%	1.90%	4.60%
Wrestling	2.60	21	0.00%	1.90%	1.20%	6.40%	4.70	14	0.00%	4.00%	2.50%	9.00%	0.40	29	0.00%	0.00%	0.00%	2.70%
Gymnastics	2.50	22	0.00%	2.60%	1.10%	4.30%	1.00	28	0.00%	0.80%	1.00%	1.60%	4.00	14	0.00%	4.20%	1.20%	8.40%
Martial arts	2.20	23	0.00%	2.50%	2.30%	1.60%	2.50	23	0.00%	3.20%	3.20%	0.90%	1.90	23	0.00%	1.90%	1.60%	2.70%
Cheerleading	2.00	24	0.00%	2.50%	1.00%	1.80%	NA						3.90%	15	0.00%	4.70%	1.90%	3.90
Frisbee	1.90	25	0.00%	1.40%	1.40%	4.10%	3.40	19	0.00%	3.00%	3.00%	5.20%	0.40	30	0.00%	0.00%	0.00%	2.40%
Field hockey	1.80	26	0.00%	1.20%	3.40%	1.80%	2.70	21	0.00%	2.60%	4.40%	1.90%	0.80	27	0.00%	0.00%	2.50%	1.70%
Lacrosse	1.80	27	0.00%	2.90%	0.80%	0.00%	2.60	22	0.00%	4.20%	1.60%	0.00%	1.10	26	0.00%	1.80%	0.00%	0.00%
Roller skating	1.80	28	0.00%	1.20%	4.00%	1.60%	2.30	24	0.00%	1.80%	5.80%	1.00%	1.30	24	0.00%	0.60%	2.40%	2.60%
Horseback riding	1.30	29	0.00%	1.80%	1.30%	0.00%	0.50	30	0.00%	0.00%	2.70%	0.00%	2.00	22	0.00%	3.30%	0.00%	0.00%
Golf	1.00	30	0.00%	1.50%	0.00%	1.00%	1.50	26	0.00%	2.00%	0.00%	1.70%	0.60	28	0.00%	1.00%	0.00%	0.00%
Ice hockey	0.50	31	0.00%	0.80%	0.00%	0.00%	1.00	29	0.00%	1.80%	0.00%	0.00%	NA					
Ice skating	0.10	32	0.00%	0.20%	0.00%	0.00%	NA						0.20%	31	0.00%	0.40%	0.00%	0.20

**Table 7:** The correlation between 1985 and 2012 PA ranking (BMI: 1 = Underweight, 2 = Normal weight, 3 = Overweight, 4 = Obese).

		BMI Category									
Inside		Boys					Girls				
Grade	Total	1	2	3	4	Total	1	2	3	4	Total
5	0.764	-	0.321	−0.800	0.000 ^b^	0.636	-	0.588	1.000 ^a^	−0.200	0.618
6	0.824	-	0.691	0.600	0.714	0.684	-	0.657	0.657	−0.257	0.670
7	0.725	-	0.517	0.262	0.643	0.698	-	0.564	0.000 ^b^	0.500	0.418
8	0.692	-	0.405	0.314	−0.333	0.573	-	0.500	0.600	1.000 ^a^	0.539
9	0.531	-	0.250	-	1.000 ^a^	0.559	-	0.393	0.000 ^b^	0.500	0.286
10	0.468	-	−0.067	1.000 ^a^	1.000 ^a^	0.429	-	−0.200	0.400	−0.257	0.491
Total	0.726	0.866	0.494	0.855	0.673		1.000 ^a^	0.532	0.545	0.308	
**Outside**											
**Grade**	**Total**	**1**	**2**	**3**	**4**	**Total**	**1**	**2**	**3**	**4**	**Total**
5	0.669	-	0.531	0.608	0.077	0.579	−1.000 ^a^	0.592	0.242	0.016	0.567
6	0.652	-	0.674	0.476	0.278	0.722	-	0.407	0.099	−0.284	0.538
7	0.562	0.257	0.406	0.503	0.262	0.492	-	0.396	0.280	0.118	0.453
8	0.530	0.707	0.440	0.514	−0.274	0.642	-	0.234	0.613	0.400	0.329
9	0.507	-	0.538	−0.060	0.631	0.423	-	0.025	−0.500	0.049	0.219
10	0.528	-	0.420	−0.714	0.683	0.563	-	0.141	0.667	0.450	0.394
Total	0.680	0.686	0.565	0.706	0.599		−0.643	0.462	0.383	0.420	

- Can not be computed because the sample is 0 or 1; ^a^ The compare sample less than three variables; ^b^ At least one of the variables is constant.

## Data Availability

Publicly available datasets were analyzed in this study. This data can be found here: https://www.cdc.gov/nchs/nnyfs/index.htm.
